# Context dependent reference states of solvent accessibility derived from native protein structures and assessed by predictability analysis

**DOI:** 10.1186/1472-6807-9-25

**Published:** 2009-04-27

**Authors:** Hemajit Singh, Shandar Ahmad

**Affiliations:** 1 Department of Biosciences, Jamia Millia Islamia, New Delhi-110025, India; 2 National Institute of Biomedical Innovation, Saito-Asagi, Ibaraki, Osaka 5670085, Japan; 3 Graduate School of Frontier Biosciences, Osaka University, Japan

## Abstract

**Background:**

Solvent accessibility (ASA) of amino acid residues is often transformed from absolute values of *exposed surface area *to their *normalized *relative values. This normalization is typically attained by assuming a highest exposure conformation based on *extended state *of that residue when it is surrounded by Ala or Gly on both sides i.e. Ala-X-Ala or Gly-X-Gly solvent exposed area. Exact sequence context, the folding state of the residues, and the actual environment of a folded protein, which do impose additional constraints on the highest *possible *(or highest *observed*) values of ASA, are currently ignored. Here, we analyze the statistics of these constraints and examine how the normalization of absolute ASA values using *context-dependent *Highest Observed ASA (HOA) instead of *context-free *extended state ASA (ESA) of residues can influence the performance of sequence-based prediction of solvent accessibility. Characterization of burial and exposed states of residues based on this normalization has also been shown to provide better enrichment of DNA-binding sites in exposed residues.

**Results:**

We compiled the statistics of highest observed ASA (HOA) of residues in their different contexts and analyzed their distribution in all 400 possible combinations for each residue type. We observe that many trippetides are more exposed than ESA and that HOA residues are often found in *turn*, *coil *and *bend *conformations. On the other hand several residues are never observed in an exposure state close to ESA values. A neural networks trained with HOA-normalized data outperforms the one trained with ESA-normalized values. However, the improvements are subtle in some residues, while they are more significant in others.

**Conclusion:**

HOA based normalization of solvent accessibility from native structures is proposed and it shows improvement in sequence-based predictability, as well as enrichment in interface residues on surface. There may still be some difference between the highest *possible *ASA and highest *observed *ASA due to an insufficiently covered space of ASA distribution in the PDB, which limit the overall improvement in prediction to a relatively modest degree.

## Background

Protein three-dimensional structure prediction directly from amino acid sequence is an important issue in bioinformatics. An intermediate approach to this problem is to predict the so-called one-dimensional structural properties of proteins. The solvent accessibility or accessible surface area (ASA) of an amino acid residue in a protein structure is one such property and the knowledge of this property can significantly enhance the overall structure and function prediction of proteins [1,2]. Given an amino acid sequence, the goal of such prediction is to estimate the ASA of each residue making use of previously observed ASA values taken from known protein structures. The knowledge from previously observed structures is modeled using machine learning and other methods [3-16]. Various methods of predicting ASA from sequence or sequence-derived evolutionary information have been developed such as neural networks [8-12], Bayesian analysis [13], information theory [14,15], multiple linear regressions [16,17], and support vector machine [18-22]. Among these, machine-learning methods such as neural networks [8-12] and support vector machines [18-22] have been shown to be the most effective for ASA prediction. Although methods to predict solvent accessibility of each atom have also been developed, more actively pursued area is to estimate residue-wise ASA [23].

In almost all ASA prediction methods, solvent accessibility is first normalized to its relative value. On the one hand, it is required for training some computational models with bound-value outputs and on the other, it gives a better idea of fractional exposure of a residue normalized by a hypothetical maximally exposed residue. Mere restricting the model outputs to finite values could have been achieved by simply rescaling all residue ASAs ignoring their identity (e.g. transforming all values by a sigmoidal function). However, the values obtained by such transformation would have little physical meaning. Moreover, the trained parameters required to model such transformed values may make the relationship between residue environments and target ASA values even more difficult to model. Thus, each residue ASA is typically normalized by its corresponding extended state ASA (ESA) values, which uses the reference state of Ala-X-Ala for normalizing ASA of residue X [24]. Thus 20 ESA values are used to normalize all residue ASAs.

We argue that this type of normalization- although better than a single value for all amino-acids, still suffers from two shortcomings. First of all, currently employed extended state of a tripeptide has no practical meaning for the residues in folded proteins and hence reference states should come from folded proteins rather than extended states. Secondly, the structural constraints imposed by actual sequence neighbors of residues are different from the case when the residue is surrounded by Ala residues on its C- and N- terminals. First of these two questions (folded context versus extended state) could be answered by using the highest values of observed ASA as reference rather than the extended state. The second question of sequence context may be answered by using 20 × 20 × 20 possible reference states instead of 20. There will still be a limitation that the highest observed ASA may not represent the highest possible ASA value due to the insufficient number of solved structures, and one has to be content with the approximations introduced by this.

Our primary benchmark in ASA normalization here is to estimate the effect of improved scaling criterion on the improvement in its prediction from sequence. An unbiased role of normalization in prediction can be assessed by using high quality data sets and developing prediction models for the two systems of normalization under similar conditions. Most non-redundant data sets of protein structures, including those used for ASA prediction are based on similarity and resolution conditions and largely ignore the incidence of missing atoms in structures [e.g. [18,22,25]]. This may be especially important for accurate calculation of solvent accessibility for each residue. To unambiguously determine the role of normalization in prediction, we develop new data sets of protein structures by systematic quality check on structures and removing samples with missing atoms. Neural networks are then trained by using two different sets of values as target vectors. Finally, the ASA values from both cases are transformed to absolute values in area units and performance comparison is made in terms of the mean absolute error and coefficient of correlation between predicted and experimental values of ASA in area units. Results indicate that HOA based normalization can improve the performance of neural network based prediction. The improvement depends on the type of residue and the score used to measure. Improvement in the correlation coefficient between predicted and experimental values reaches to as high as 10% using HOA instead of ESA for normalization. We also demonstrate that this type of normalization may be effective in estimating interface residues simply from their over-exposed status.

## Results and discussion

### Distribution of context-dependent ASAs

**Figure 1:**
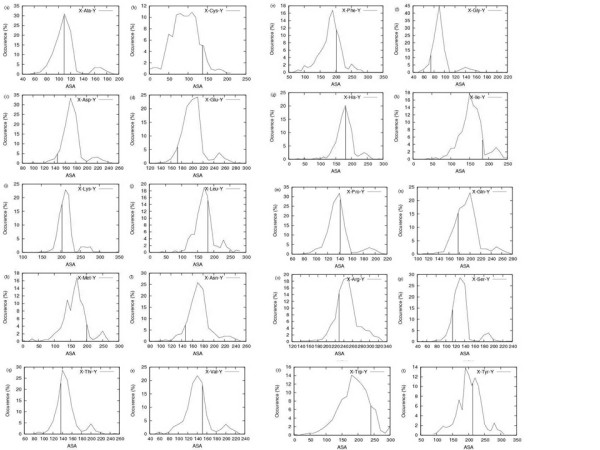
**Histogram of highest observed ASA (HOA) of X-Z-Y tripeptides for residue Z where X and Y are the flanking amino acid residues in actual structures**. The Vertical line indicates (currently used) extended state ASA (ESA) of Ala-X-Ala. Subplots are arranged alphabetically by one-letter code (Ala to Tyr). X-axis shows the HOA and Y-axis shows the number of tripeptides (out of 400 possible combinations), whose HOA falls in that range.

Figure 1 (a-t) show the distribution histograms of highest observed absolute values of ASA (HOA) of residues in native protein structures taken from PDB for each possible tripeptide environment (21 × 21 possibilities; 20 for amino acid types and one for the absent neighbor in the terminal positions) (note that only structures of high quality without a missing atom are counted). Figure S1 (Additional file 1) provides additional statistics by superimposing ASA distribution of residues on their corresponding HOA values. These graphs clearly indicate that the highest value of ASA attained by a residue strongly depends on the residue neighbors on its C- and N-terminals. Clearly in a large number of cases a higher ASA is observed for at least some tripeptides than what is obtained from Ala-X-Ala extended state values. Main observations are summarized as follows:

#### Many tripeptides have higher ASA in folded state than the Ala-X-Ala extended state

**Figure 2:**
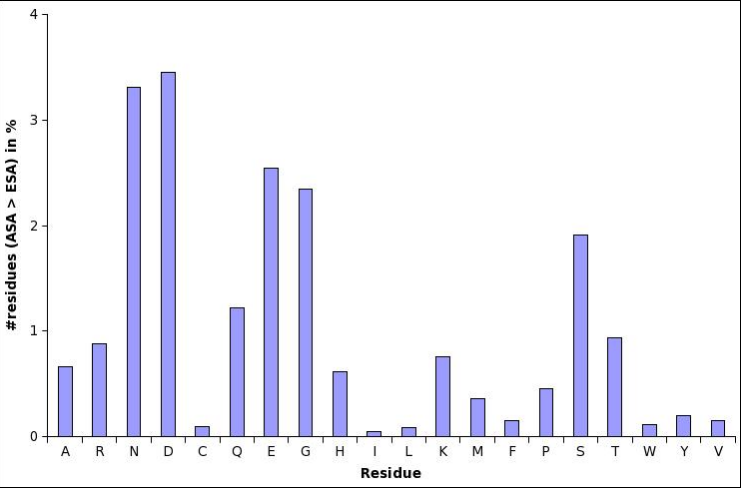
**Percent residues with more than 100% ASA values, if normalized by their extended state ASA (ESA)**.

Extended state ASA has been used for normalization under the assumption that it represents unfolded state of the protein and would probably be more than any observed ASA in real proteins. However, as seen from Figure 1, the extended state ASA (shown by dropped lines in plots) are not on the extreme right in any of the plots. Thus, for each residue there exist a number of tripeptide environments in which their ASA can exceed Ala-X-Ala ESA values. Figure 2 shows the fractional number of residues whose ASA is more than the extended state ASA as described above. Detailed numbers are provided in Table 1. Clearly there are a significant number of such over-exposed residues, especially with polar or charged side chains. This result is potentially of interest for computing free energy of denaturation, but we limit ourselves to the effect on sequence-based predictions.

**Table 1:** Number of over-exposed residues with their exposed surface area (ASA) greater than the Ala-X-Ala extended state ASA (ESA).

**Res.**	Freq (%)	**Res.**	Freq (%)	**Res.**	Freq (%)	**Res.**	Freq (%)
**A**	0.66	**G**	2.35	**M**	0.36	**S**	1.91
**C**	0.10	**H**	0.62	**N**	3.31	**T**	0.94
**D**	3.45	**I**	0.05	**P**	0.45	**V**	0.15
**E**	2.54	**K**	0.76	**Q**	1.22	**W**	0.11
**F**	0.15	**L**	0.09	**R**	0.88	**Y**	0.20

Typically, polar and charged residues have a significant number of such cases.

#### In some tripeptides ESA values are never observed

Figure 1 shows that all residues in general but the hydrophobic ones in particular (e.g. Trp) have several tripeptide contexts which lie to the left of dropped line showing ESA values in their plots. This means that there are many tripeptides environments in which the central residue remains in a partially buried state, either due to a folded nature of the tripeptide or due to inevitable long range contacts. This argument is limited by the fact that some of the highest observed ASA (HOA) values may not represent the actual highest *possible *ASA, because the structures showing them more exposed may eventually be solved in future.

#### HOA histograms of some residue types have sharp peaks

Some residues such as His, Lys and Ser show a sharp peak in their histograms and most HOA values are close to ESA values. This suggests that the highest exposure these residues can have, depends only weakly on their neighbors and HOA-normalized values will at best rescale the ESA-normalized values in these cases. On the other hand residues such as Cys and Trp have less sharp peaks in their histogram, showing that the highest ASA of these residues strongly depends on their sequence neighbors. However, flatness of histograms is also caused by a low frequency of these residues in protein structures. It remains to be explored if these residues will continue to have leptokurtosis in their histograms when more data on their tripeptides becomes available.

Observation of ASA values higher than ESA has led in the past to relative ASA being more than 100%. Using HOA-normalization, relative values will never cross 100% and may be more suitable for using a machine learning method for prediction. However, the tripeptide data may be modified when more data becomes available and the results presented here may also need minor revisions with more solved structures.

#### Correlations between HOA- and ESA- normalized values are strong with subtle differences

Table 2 shows the coefficient of correlation between relative ASA values obtained by HOA and ESA methods. Clearly, the differences are subtle as shown by high correlations. Coefficient of correlation will be higher if HOA distribution has a sharp peak, even if it is higher than ESA values (as in Asp and Glu), because the role of HOA-normalization in such a case will be simply to rescale ESA values. However, in more flat distributions, correlation is affected because of different values of HOA used for normalization in various contexts (neighboring residues). A larger variation of HOA values in various tripeptides is either caused by a relatively smaller number of observations available in that residue or greater role of structure and neighbors. However, lower correlation cannot be entirely attributed to the fewer data points in all cases as we observe differences between ESA and HOA even for identical Ala-X-Ala environment, which are sufficiently well-populated. Thus, at least some contribution to lowered correlation comes from structural constraints in real proteins.

**Table 2:** Frequency of residues in Ala-X-Ala conformations, their extended state ASA (ESA) values and highest observed ASA (HOA) obtained from the entire data set of proteins (8.9 million residues, overall including residues with different sequence neighbors).

**Res**	Freq	ESA	HOA	CC	**Res**	Freq	ESA	HOA	CC
**C**	998	140.4	120	0.9200	**L**	7428	183.1	173	0.9885
**W**	889	240.5	217	0.9633	**S**	3694	117.2	142	0.9885
**M**	1554	200.1	181	0.9740	**T**	3049	138.7	149	0.9891
**Y**	1857	213.7	223	0.9813	**Q**	2810	178.6	204	0.9893
**F**	2453	200.7	202	0.9838	**N**	2910	146.4	176	0.9895
**I**	4114	185.0	167	0.9843	**R**	3297	229.0	280	0.9895
**H**	1849	181.9	200	0.9845	**P**	2528	141.9	138	0.9901
**G**	5328	78.7	96	0.9857	**K**	4192	205.7	219	0.9916
**A**	8032	110.2	120	0.9873	**E**	4962	174.7	201	0.9917
**V**	5704	153.7	148	0.9875	**D**	4567	144.1	165	0.9934

Correlation coefficient (CC) between relative ASA obtained after normalizing by the Ala-X-Ala extended state (ESA) and tripeptide highest observed ASA (context-dependent ASA) is also provided in the last column. CC values are based on all residues with different sequence neighbor. Residues are arranged in the order of CC values.

Abbreviations: Res: residue ID, Freq: number of residues in Ala-X-Ala conformation, ESA: extended state ASA of Ala-X-Ala conformation, HOA: highest observed ASA in Y-X-Z conformation (any neighbor), CC: correlation coefficient between ESA-normalized and HOA-normalized values in the benchmark data set.

Further statistics and implications of even these small differences in HOA and ESA-normalized values are examined in the following sections.

#### Overall ASA distribution of a tripeptide

**Figure 3:**
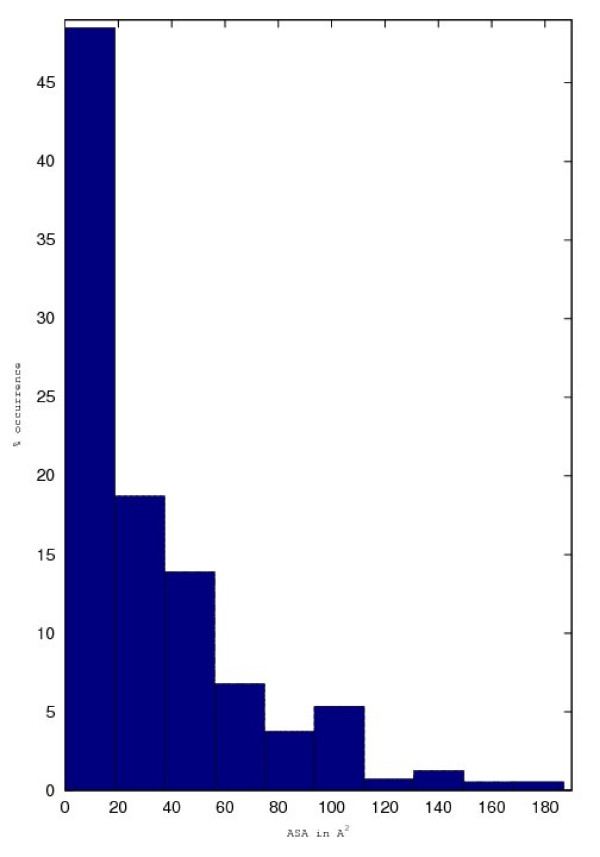
**Typical distribution of ASA for a residue in similar tripeptide environment**.

Although, we are looking for the highest observed ASA in tripeptides, we also analyzed the distribution of ASA within all tripeptides of a given type. A typical distribution is shown in Figure 3 and it is observed that the tripeptides have ASA diversity similar to the overall ASA distribution of all residues.

#### HOA residues primarily come from coil, turn and bend conformations

**Figure 4:**
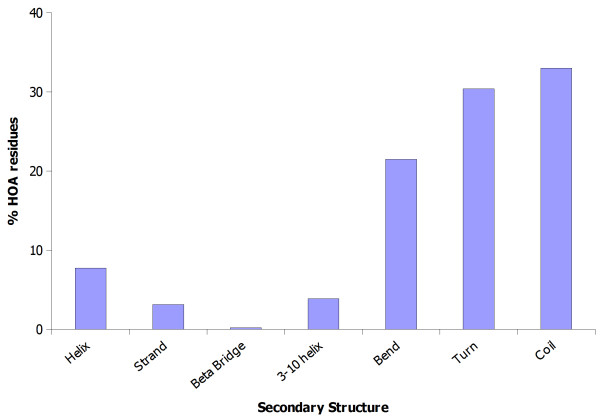
**Distribution of residues' trippetide environments with highest ASA in various secondary structures and**.

Table 3 shows the distribution of HOA residue environments into various secondary structure types. They are also plotted in Figure 4. We observe that 33% HOA residues occur as *coil*, whereas ~30% are in *turn*, whereas ~22 are in *bend *conformations. This can be understood in terms of folding patterns because these three conformations allow more atoms of the residue to be exposed and could also be a reason why many of them have higher than the extended state ASA values. Thus, the role of structure is not only to restrict the exposed surface of a residue but also to over-expose some them.

**Table 3:** Distribution of 8000 HOA residues environments in various secondary structures.

**Secondary** **Structure**	**% HOA residues**	**Number of HOA residues**
Alpha-helix	7.73	672
Strand	3.11	271
Beta Bridge	0.21	18
3–10 helix	3.86	336
Bend	21.50	1874
Turn	30.40	2650
Coil	33.00	2870

For each tripeptide environment, (central) residue with highest ASA is selected and distribution of such residues in various secondary structures is calculated.

#### Why do HOA values differ from ESA values?

HOA values obtained in the current work differ from the traditional ESA values in the following ways.

(i) Actual protein environment instead of a purely computational extended state is used in our proposed method. Traditionally Ala-X-Ala or Gly-X-Gly environments are generated in simulation software. These software programs produce a hypothetical state of a tripeptide, which may never actually be observed in a protein or even a fully capped tripeptide. In our approach, we scan the entire set of observed tripeptides in actual crystal structures and the effect of solvent, charge screening and the effect of subsequent peptide bonds on neighbors are implicitly taken care of. Thus the new approach of finding a normalization value is more realistic than currently used method.

(ii) Instead of assuming Ala residues on both sides, a detailed residue context is used that allow for taking care of additional constraints as well as potential role in over-exposing some of the residues in a given context.

### HOA from native structures versus molecular calculations

Usual practice to estimate the highest possible ASA of a residue is to carry out molecular calculations assuming simple aliphatic side chain neighbors on its N- and C-terminal positions. As stated above, we depart from the conventional methods by (i) choosing native protein structures instead of simulated tripeptides to address the role of global protein environment and (ii) considering N- and C- terminal context by obtaining 8000 reference states rather than currently available 20. In making these two departures, following questions may rightly be raised.

(i) Are there sufficient examples in PDB sto obtain highest observed ASA (HOA) close enough to highest possible ASA (HPA) for each tripeptide environment?

(ii) Is there any advantage of using native protein structures over conformations derived from molecular simulations of tripeptides?

We discuss these issues in the following.

#### Is there sufficient data for obtaining HOA values?

As stated above, Highest Observed ASA (HOA), analyzed here may be different in some cases than the Highest Possible ASA (HPA), because the ensemble size formed from the data set may not have sufficient number of representatives in the protein structures solved so far. This insufficiency has partly been the reason that extended state has been generally used as a reference state. To address this concern, we first note that the overall number of residues from which HOA values have been extracted here is ~8.9 million (the number of residues on which the effect has been analyzed is 376000). This is a sufficiently large data size and if some of the 8000 tripeptide patterns have not shown up sufficiently in HOA ensemble of millions of residues, they must be indeed rare and that is unlikely to affect the results of current analysis. We only use HOA of a residue for normalization if the HOA query for that tripeptide was based on a minimum number of observations in the universal ensemble of tripeptides (actual number in the predicted data is likely to be much rarer). To estimate the effect of insufficiency of HOA search space, we present some additional statistics as follows.

Of all the 8000 tripeptide patterns, more than 97% occur at least 100 times in the overall search space (OSS). A frequency of 100 in OSS corresponds to only a few occurrences (~4 for each of these tripeptide patterns) in the normalization benchmark dataset (NBD) whose size (~376000) is about 4% of OSS. It may be noted that occurrence of 4% of 8000 patterns does not mean that there are anywhere close to 4% residues which were normalized by infrequent pattern HOA data because 4% refers to 8000 possible tripeptide patterns and rarer of them are even rarer in the NBD dataset as 96% of 8000 residue patterns are far more degenerate than are these 4% cases. Thus, although HOA values for very few tripeptides are based on a small number of observations, it is unlikely to affect the results of the current study. The statistics will also be refined from time to time to take into account if any newly observed ASA of a tripeptide suprpasses its currenly listed HOA value.

#### Is there any advantage in using native conformations over simulated structures?

**Figure 5:**
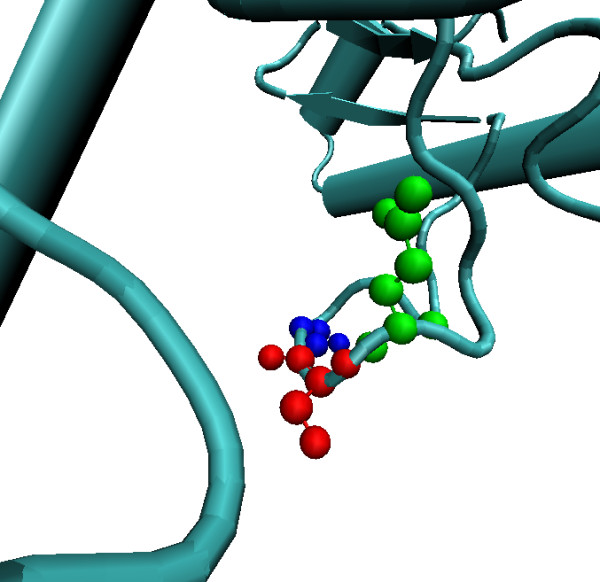
**A Ser residue in *turn *conformation, surrounded by Gln and Gly on N- and C- terminals respectively, making it highly exposed (observed ASA = 142 Å^2 ^, ESA = 117.2 Å^2^)**.

There are several advantages of using native-structure reference states rather than simulated structures. First of all molecular simulation of a tripeptide requires definition of a conformation. Extended tripeptide state has been assumed to be the most exposed conformation presumably because it is expected that any folding should reduce the ASA of a residue. However, we observe that number of residues may be significantly more exposed in native proteins than their extended state. For example Figure 5 shows a Ser residue to be more exposed in Gln-Ser-Gly conformation (ASA = 142 Å^2^) compared with its extended state (ASA = 117.2 Å^2^) in Ala-Ser-Ala derived from molecular simulations. Such observations of higher ASA than ESA are frequent as discussed throughout this work. However, to establish that higher ASA is not due to different C- and N-terminal residues only, we compared 20 ESA values of Ala-X-Ala tripeptides with their corresponding 20 HOA values (see Table 2). It is observed that in 13 of the 20 cases we did observe an ASA of a residue even in identical Ala-X-Ala environment, which was higher than its corresponding extended state ASA. This shows that the native structure not only constraints a residue's ASA but also makes it more exposed in certain conformations. Out of 7 remaining residue types Pro and Val, show a difference of less than 10 Å^2 ^but in six cases (residues: Cys, Ile, Leu, Met and Trp) HOA's are always significantly lower than ESA values despite a sufficiently large number of their occurrence (998, 4114, 7428, 1554, 889) respectively and it remains doubtful (partly due to their hydrophobic nature) that they will ever be observed close to their ESA conformation in real proteins. Thus native state HOA based reference state carry information which would not be available from structures generated by molecular simulations.

### Implications to ASA Prediction

**Figure 6:**
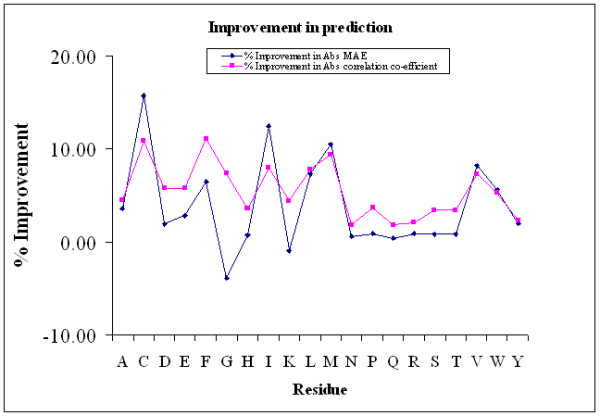
**Improvement in the prediction of ASA using HOA- and ESA- normalized data sers**. Both MAE and correlation co-efficient between absolute ASA values are shown and improvement is defined relative to the MAE in the ESA-normalized predictions.

Table 4 reports the main results of prediction and the results of a Student's *t-test *to assess the statistical significance of the improved performance. Also provided are results of *p-values *obtained by *Mann-Whitney *statistical significance test (*u-test*). Results of the two tests are generally similar but *t-test *was found to label fewer residue cases to be significant and hence discussions are based on these values. Graphically, the results are seen in Figure 6. Mean absolute error (MAE) and coefficient of correlation obtained by two types of normalization are compared. Number of residues in the database is also shown. In terms of relative ASA, the prediction performance is significantly higher in case of some residues (e. g. in Asp: 17.3% for HOA, 21.04% for ESA). However, true difference in the performance of relative ASA cannot be directly compared because HOA may render overall range of ASA to be lower than ESA-normalized values. Since, buried residues are generally better predicted; ASA normalized to smaller values may give a wrong impression of improved performance. Therefore, to evaluate the difference in performance caused by normalization, predicted values are reconverted to absolute ASA and the difference in the performance is compared (see Methods). Last two columns of MAE and correlation parts of the Table 4 and all p-values are based on these results. It is observed that there is a small but clear improvement in prediction performance of data trained using HOA-normalized ASA. The difference is of the range of only 1 Å^2 ^but statistically significant. In terms of coefficient of correlation, the difference is more clearly visible. For example Cys residue ASA correlation improved from 0.33 to 0.37, Phe residue improved from 0.40 to 0.45. Compared to the correlation between predicted and experimental values the improvements are close to 10% in several cases. This highlights the importance of more accurate normalization procedure in prediction of ASA. An analysis of p-values in Table 4 shows that the improvement in prediction is most significant in hydrophobic residues such as Ala, Cys, Phe, Ile, Leu, Val and Trp. Another residue Met also shows a significant improvement as it often occurs in terminal positions and HOA takes care of this fact much more neatly than ESA-based normalization. Polar and charged residues such as Ser, Arg and Asn show insignificant improvements, as they are generally more difficult to predict. Unusual amino acid Pro also shows poor improvement, probably because not enough number of residues was observed to assign HOA values with confidence.

**Table 4:** Comparison of the prediction performance obtained by using ESA- and HOA-normalized target ASA values.

**Res.**	**Freq**	**MAE**				**CC**				**P-values**	
							
		Rel (ESA) (%)	Rel (HOA) (%)	Abs (ESA) (Å^2^)	Abs (HOA) (Å^2^)	Rel (ESA)	Rel (HOA)	Abs (ESA)	Abs (HOA)	t-test	u-test
A	30972	14.81	14.13	16.22	15.64	0.63	0.65	0.63	0.66	0.002	5.0e-10
C	5156	10.39	13.28	14.53	12.25	0.33	0.35	0.33	0.37	8.39e-13	0
D	22482	21.04	17.30	30.31	29.73	0.49	0.50	0.49	0.52	0.018	0.579
E	25303	20.09	17.00	35.11	34.12	0.49	0.50	0.49	0.52	5.21e-04	0.027
F	15016	11.60	11.63	23.22	21.71	0.40	0.44	0.40	0.45	7.28e-06	0
G	27935	20.25	17.60	15.31	15.90	0.51	0.52	0.50	0.54	0.621	2.3e-4
H	8596	16.22	16.36	29.50	29.28	0.54	0.55	0.54	0.56	0.825	0.581
I	21345	10.80	11.21	20.05	17.55	0.46	0.50	0.46	0.50	2.79e-13	0
K	21997	17.82	17.46	36.64	36.97	0.43	0.43	0.43	0.45	0.015	0.008
L	34119	11.40	11.27	20.86	19.34	0.47	0.51	0.47	0.51	1.58e-12	0
M	8130	12.20	12.99	24.40	21.84	0.48	0.52	0.48	0.53	3.44e-10	0
N	16660	20.87	17.63	30.55	30.38	0.53	0.52	0.53	0.54	0.621	0.148
P	17214	17.96	18.39	25.47	25.23	0.52	0.53	0.52	0.54	0.514	0.426
Q	14334	18.73	17.21	33.46	33.31	0.52	0.52	0.52	0.53	0.993	0.194
R	18302	17.57	16.21	40.23	39.85	0.46	0.46	0.46	0.47	0.243	0.647
S	22408	18.62	16.48	21.83	21.64	0.56	0.57	0.56	0.58	0.944	0.092
T	20973	16.71	15.61	23.17	22.97	0.56	0.58	0.56	0.58	0.710	0.343
V	26399	11.72	11.69	18.04	16.56	0.52	0.55	0.51	0.55	0.000	0
W	5625	11.77	13.80	28.35	26.76	0.36	0.37	0.36	0.38	0.008	9.93e-7
Y	13636	13.24	13.45	28.29	27.73	0.42	0.43	0.42	0.43	0.110	0.040

Abbreviations: Res: Residue ID, MAE: mean absolute error in prediction measured in percentage points, CC: Correlation coefficient, Rel: relative ASA values, Abs: using absolute area units, t-test: Student's t-test statistics, u-test: Mann-Whitney u-test

We also compared the mean absolute error in absolute ASA prediction for data corresponding to each of the 8000 tripeptide and found that in 5220 (65.3%) cases mean absolute error of HOA-normalized tripeptides was lower compared to 2771 (34.6%) cases in which HOA-normalized tripeptide prediction error was higher (9 cases showed no difference). This shows that there are many more tripeptides contexts in which prediction performance is improved by using HOA-normalization, than those whose performance fell (apparently due to noise in the prediction model). Test of significance on individual tripeptides is not possible because the prediction is performed on a (smaller) non-redundant data set of proteins. There are about 376000 residue-wise predictions for 8000 patterns implying ~47 instances per tripeptide type on the average. Given that prediction itself is not 100% accurate and prediction errors have large standard deviations, it is not possible to detect statistical significance in the differences between prediction performances for each of these tripeptide patterns.

### Application to interface prediction

**Figure 7:**
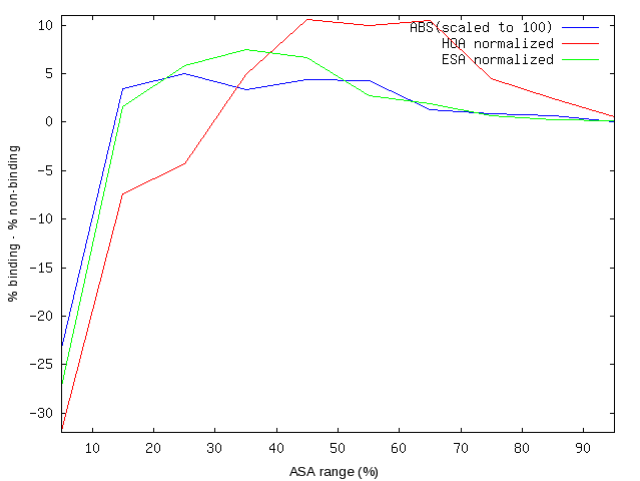
**Difference between the frequency of DNA-binding residues and non-binding residues in various ASA ranges taken from protein-DNA complexes**. This difference is best separated for buried and exposed regions defined in terms of HOA-normalized relative ASA. According to that classification, we can say with the best confidence that exposed regions contain many more DNA-binding residues than buried regions.

The aim of the present work is to analyze the statistics of highest observed ASA of residues in proteins and its implications to prediction of ASA itself. However, it is useful to see if the newly defined relative ASA values carry any more physical meaning than what is already available in relative ASA obtained by ESA-based normalization. The most important meaning of relative solvent accessibility (RSA), in contrast to absolute accessible surface area (ASA) is to characterize them between exposed and buried residues at given cutoffs. One of the most important applications of this characterization is to determine a relationship between ''unusual exposure'' and ''interface propensity'' [26,27]. Based on this characterization, it is expected that the exposed residues contain a greater fractional number of binding sites than the buried ones. We examined if such a distinction could be improved by the proposed method of normalization. Figure 7 shows a graph of the difference between fractional number of binding and non-binding residues in various ranges of RSA based on ESA and HOA definitions of normalization. For comparison a third plot based on absolute ASA, scaled to 100 has also been shown. Binding sites data is as reported by us in our previous works [28]. From this graph a clear improvement is seen in the enriched number of binding residues in the exposed regions defined by the HOA normalization. This demonstrates another useful application of fine-tuning the RSA calculations as described in this work.

### Availability

A perl program to normalize ASA values by the proposed method has been provided for download on the web at . This program converts absolute ASA values to HOA- or ESA-normalized values and vice-versa. Users can also provide their own HOA data, which enables a quick update or return to ESA values for some of the tripeptides. HOA data will be regularly updated if higher ASA values are observed for a new tripeptide.

## Conclusion

In this study, we developed the statistics of highest observed ASA in various tripeptide environments of residues. Using ASA data normalized by these ASA values, we could predict ASA with ~15% MAE and 0.67 correlations from evolutionary information. Individual residues show varied degrees of improvement in their prediction when trained with data normalized by new method. We also show that the exposed regions defined by newly developed method of normalization are better enriched in binding sites for the DNA-binding proteins. It remains to be seen, if the proposed method of normalization has other universal applications, although the present observations suggest that trend.

## Methods

### Datasets

Solvent accessibility information about protein structures were directly taken from DSSP database available on the web . From all the available 37,964 entries in DSSP database at the time of starting this work (October 2006)), we removed those whose coordinate files in PDB had some missing atoms and whose resolution is poorer than 2.5 Å. All residues were checked for completeness to ensure the quality of tripeptide ASA data. This resulted in 18,758 structures, all of which were used to obtain highest observed values of ASA for each tripeptide. This data set consists of more than 8.9 million residues and hence an identical number of tripeptides. We call this data set overall search space (OSS) from which highst observed ASA (HOA) is extracted.

For the purpose of evaluating predictive performance, we used a dataset taken from the protein sequence culling server PISCES with sequence identity less than 25% and X-ray resolution of 2.5 Å [25]. This dataset consists of 4478 protein chains. The chains with missing coordinates, unknown structure regions and length less than 30 amino acids were removed by an in-house program. Further, known membrane protein chains were also removed from the dataset. This resulted in 1708 proteins chains. DSSP program was used to calculate the residue solvent accessible surface area for a given protein structure [29]. This dataset contains about 376000 residues and is called normalization benchmark data set (NBD).

### Residue context normalization values

Tripeptide patterns of solvent accessibility look similar to our 1P1N patterns of the look up tables, which we developed for predicting ASA [30]. In this method, ASA of a residue was assigned by taking the n-peptide environment of a residue from query sequence and then scanning a previously compiled database of such n-peptides ASA values. The database to be scanned is called a lookup table or ASA dictionary and a residue's tripeptide environment is defined as 1P1N, 2P2N etc. (P for previous and N for next neighbor). 1P1N refers to a tripeptide environment. However 1P1N dictionaries consist of mean observed values in tripeptides, whereas we are interested in the highest observed ASA value here. Thus, there are 20 types of residue, which may be preceded by any of these 21 environments (20 amino acid residues or case of absent neighbor in a terminal). So, there are 21 degrees of freedom on the location preceding and an equal number of choices are possible for residue followed by next neighbor. Some of these patterns have a very low frequency in the entire DSSP database. So, for our analysis and prediction, we considered the patterns which occurred more than X times in our sample. ASA of all other residue contexts in which corresponding normalization cannot be performed in the newly proposes system (due to insufficient data), were excluded from analysis. Value of X was taken as 30 in the current study. Although values higher than 30 were tried to have a reasonable number of tripeptide patterns as well as to have enough number of data in each category, this number was found to be in intuitive balance.

### Normalization from the dictionary

To make a normalization of ASA for a residue, we start looking up in the tables for a pattern. For example, if we want to normalize ASA for Ala in sequence where Ala occurs as Gly-Ala-Ser, the normalization will start with a search for G-A-S pattern in the table. If the pattern is present in that table, we normalize the ASA of Ala by that pattern. If the pattern does not exist in the dictionary, we go to the previous normalization method of Ala-X-Ala [11] or exclude it from the analysis.

### Neural network details

This work primarily aims to study normalization method and therefore an established and widely used protocol for predicting ASA has been used. Thus, based on many published methods, including ours [e.g. [1,8,9,12]], evolutionary information, amino acid composition and protein chain length are the descriptors used for the prediction model. Effort is not made to better the existing best performance for prediction, but to generate a simple reproducible model with identical inputs to the two normalizing methods, so that the role of normalization can be established.

This may be noted that normalizing ASA by HOA values prior to forming target vectors may be regarded as giving some kind of residue neighbor information to the feature vectors. However, residue neighbor information is provided in any sequence-based prediction implicitly anyway and although the improvement in performance seen here could be due to this explicit availability in the initial weight matrix, the fact remains that such normalization improves model performance.

#### Evolutionary Information

Evolutionary information, forming the input vectors for the prediction model, was generated using the program PSI-BLAST [31]. E-value cutoff for this purpose is 0.1 and similar sequences are searched in the non-redundant protein sequence database (NCBI NR database) to build the multiple alignments. Three iterations of PSI-BLAST were performed; no masking of low complexity regions or membrane domain was used. The alignments were represented as profiles or position-specific substitution matrices (PSSMs). PSSM rows provide the log odd frequency of occurrence for the 20 amino acid residues at each position of the sequence. In positions, where similar sequences are not observed or if no other residue occurs in given position of aligned sequences PSSM row is simply the corresponding entry for that residue type in BLOSUM62 substitution matrix. PSSM data obtained from BLASTPGP were directly used as inputs to our feed-forward neural network, consisting of an input, an output and a hidden layer.

#### Design and Training

There are 20 units for each residue from PSSM. In order to allow a window to extend beyond the N-terminus and the C-terminus, a special null indicator was added for each residue. The protein sequences were presented to the neural networks as windows, or subsequences, of 17 residues including the amino acid of interest, which slide along the entire sequence. The total number of windows or patterns for a particular protein is therefore equal to the number of residues in the protein. Additional information of amino acid composition and chain length were also presented as input vector. Therefore, each input vector size is be 21 × 17 + 20 +1 = 378 units. The Stuttgart Neural Network simulator (SNNS) version 4.2 package with default setting of BP algorithms was used to train a fully connected, feed forward neural network [32]. The network architectures discussed involve an input layer consisting of nodes equal to the number of input vectors, hidden layer consisting of three nodes and one output layer consisting of a single node.

### Measurement of prediction performance

We adopted the same measurements used in our earlier works [11]. They are reproduced for quick reference.

#### Mean absolute error (MAE)

MAE of prediction is defined as the per residue absolute difference between the predicted and experimental values of ASA, which can be express as:

(1)

Where the summation is carried out for all residues and N is the number of residues in the entire data. MAE is measured in percent units for relative ASA and in Å^2 ^for absolute values. O and P refer to observed and predicted values of ASA and ABS indicates that the absolute errors are considered.

#### Pearson's correlation coefficient (CC)

It is also another important indicator of prediction performance, which can be calculated by:

(2)

Where *o*_*i *_and *p*_*j *_are the experimental and predicted values of relative solvent accessibility, respectively.

#### Performance improvement

Improvement in prediction performance is measured by the following expression.

(3)

Where MAE(HOA) and MAE(ESA) refer to the mean absolute error obtained by HOA- and ESA-normalized method respectively. MAE itself refers to relative or absolute ASA depending on which improvement is being measured. MAE is replaced by coefficient of correlation, when comparing performance by that score.

### Validation Method

Three fold validation methods were carried out. The whole dataset was randomly divided into three approximately equal parts. Training was done on two-thirds of the data and testing on the remaining third. After running this process three times, an average of MAE and CC, over all the three test datasets was calculated and is listed in the results tables (Table 4).

### Statistical significance of difference in prediction performance

Improvement in prediction performance is obtained by comparing mean absolute error of prediction. However, since there are several types of normalization considered, we reverse-transform all predicted and observed values to absolute area unit before comparison. Thus the absolute error in absolute ASA prediction error is used as a measure of performance in such comparisons. Overall performance of neural network trained on a given normalization scheme is given by (1) such that ASA refers to absolute area units in such comparisons. Reverse transformation to absolute units ensures that the comparisons of prediction performance are carried out on the same scale. Absolute error in each residue ASA is computed and p-values are obtained between the statistics of two error-distributions, for which comparison is made. A Student's t-test is used to assess the statistical significance of difference between these two distributions. Since, the error distributions are generally not normal, an additional test of significance was performed, by using Mann-Whitney's u-test, which gave largely similar results. Both tests of significance were carried out using modules in open source programming language *Octave *.

## Authors' contributions

This work is part of the doctoral research of YHS. Most calculations were performed by HS under SA's guidance. SA designed experiment, analyzed results and led the manuscript preparation.

## Supplementary Material

Additional File 1 **Additional statistics and data sets**. Details are provided in Readme file included in the compressed file. Click here for file
